# Collectanea

**Published:** 1829-03

**Authors:** 


### COLLECTANEA.

Floriferis ut apes in saltibus omnia libant,
Omnia nos, itidem, depascimur aurea dicta.

### PHYSIOLOGY.

1. *On Seeing in Water.*—THOSE animals whose eyes are organized for seeing in water, see but indifferently in air. Hence, in those cases where the habits of the animal require it to see in both media, it is provided with two sets of eyes, or with eyes accommodated for seeing in each element. Thus the Gyrinus natator, an insect which generally swims on the surface of the water, but half submerged, is provided on each side with two eyes, one pair situated on the crown of the head, for seeing in the air, and another pair under the head, for seeing in the water. It is also probable that the fish named Cobitis anableps, which has in each eye an upper and under cornea of different curvatures, and for each cornea a particular anterior surface of the lens, is capable of seeing in water with the one half of the eye, and in air with the other half. Thus SOEMMERING found in this fish, the semidiameter of the upper cornea = 1.0; the under = 1.2; the two curvatures of the upper part of the lens = 0.5; and the two curvatures of the under part of it = 0.2 Paris lines. It cannot be denied that, in general, land animals can see under water, and aquatic animals in air: even man sees under water, although the contrary has been maintained. It is not, however, possible that the same eye is ever so organized as to see equally well in both elements. Land animals always see indifferently in water, and aquatic animals imperfectly in air. The one is long-sighted in water, and the other short-sighted in air. An animal in which the eye is adapted for seeing equally well in air and water, can have but imperfect vision in either. These conclusions are in conformity with what is known of the power of vision in those animals that live partly on the land and partly in the water. The seal (Phoca) is one of those animals that live in both elements; but the seal has but imperfect vision in the air. ROSENTHAL, in his Memoir on the Organs of the Senses in the Seal, says, "We have convinced ourselves by careful observation on living seals, of the species Phoca grypus of FABER, that the animal is always short-sighted in the air; for when we held before it fish and other bodies, as pieces of wood or stones, it did not distinguish them accurately, until they were brought so near that the organ of smell could be called into activity." SCORESBY remarks, "Whales are observed to discover one another, in clear water, when under the surface, at an amazing distance. When at the surface, however, they do not see far." (*Scoresby's Arctic Regions*, vol. i. p. 456.) FABER, in his very interesting work on the habits and manners of birds that inhabit high northern latitudes, (p. 298, § 55,) remarks that divers (Colymbus) do not see so well above water as grebes (Podiceps), but better under water, because it is there that they obtain their food.

It also appears that birds which see well in one element, do not see so well in the other. Faber proposes the question, "Is it the case that divers, when under water, draw their nictitating membrane over the eye, as they do when looking towards the sun, in order to prevent the contact of the water!" It would appear, from the observations of TREVIRANUS, from whose excellent work, entitled "Beiträge zur Anatomie und Physiologie der Sinneswerkzeuge des Menschen und der Thiere, von Dr. G. R. Treviranus; fol. Bremen, 1828," the observations on vision we are now detailing are principally extracted, that, by drawing the nictitating membrane over the eye, divers, and all other land animals which seek their food under water, are enabled not only to prevent the immediate action of the water on the eye, but also to discover their prey. But, as the light loses more of its power on passing through water than in passing through air, and is still more weakened in its progress through the nictitating membrane, it follows that, owing to this membrane, vision must be less distinct under water than in the air.

2. *How do some Animals see in the Dark?*—Is there any arrangement in the eye, and what is it, by which animals that see in the dark are enabled to make up for the want of external light? When we consider the metallic lustre of the tapetum, which in many animals occupies a great part of the choroid coat, or even its whole surface; further, its resemblance to a concave mirror, and its relation to the light that penetrates into the interior of the eye, we cannot help considering it as the means employed for this purpose, by its collecting the light, and illuminating, by its reflection, objects lying in the axis of the eye. PREVOST objects to this explanation, that there are many animals whose eyes have no tapetum, although they conduct themselves as if they saw in the dark. This is actually the case. The tapetum occurs in carnivora, ruminantia, pachydermata, cetacea, owls, crocodiles, snakes, rays, and sharks: it is wanting in man, apes, glires, chiroptera, hedgehogs, and moles; in birds, with exception of owls; and in osseous fishes. But the gnawers or glires, bats, the hedgehog and mole, are animals that obtain their food more by night than during the day; and many of them conduct themselves in the deepest darkness as if they were directed by the sense of sight. But this objection may be obviated by remarking, that it is probably some other sense than that of vision which procures for many of these animals sensations of external objects in the dark. We have in favor of this opinion not only the experiments of Spallanzani on bats, from which it appears that, after these creatures were deprived of the use of their eyes, they conducted themselves as if they still possessed the power of vision, but also the examples of species of that family, in which the eyes are so imperfectly developed, or lie so much concealed behind the outer skin, that it is of little or no use to the animal. The genera that see in the dark have undoubtedly so irritable a retina that they can only see during a very feeble light; whereas, in those animals whose eyes are organized equally for daylight and nocturnal darkness, the retina possesses less irritability. Hence, although these are without a tapetum, it does not follow that this organic part does not afford a mean for seeing during a feeble light.

The tapetum is either spread over the whole choroid or only over the upper half of it. The first is the case with the cetacea, owls, and with those amphibia and fishes which are provided with this shining envelope; the second occurs in carnivorous and ruminating animals. It is more extended in the ruminating than in the carnivorous tribes. But it always extends so far as to encompass the posterior extremity of the internal ocular axis. All the rays of light from external objects which reach it are united on it, through the transparent part of the eye, and it again reflects back the whole united rays towards the lens. This latter unites them into a single cone, which has the ocular axis as its axis, and its point is directed outwards. The very convergent rays of this cone become more divergent by their passage from the lens into the aqueous fluid, and from this into air or water. Finally, the apex of this cone falls into the point of the most distinct vision; for in this point is situated the focus of all the rays that reach from the interior of the eye to the posterior surface of the lens. The cone is complete when the tapetum is spread over the whole of the choroid; but the upper half of it is wanting when it occupies only the upper hemisphere of this coat. The tapetum is confined to the upper half of the choroid in all animals whose residence and manner of life are of such a nature that the under half of the retina is immediately struck by bright daylight; and for this simple reason, because the animal must have been dazzled by the reflection of the bright light from the under half of the latter. It covers the whole posterior portion of the internal eye in the cetacea and owls, many amphibia, rays, and sharks, because these animals live constantly in the water, or in a feebly luminous medium, or have their place of residence in dark corners, or go in quest of food during the night.

The experiments and observations of Prevost and Esser show that the reflection of light from the tapetum is the cause of the luminousness of the eyes observed under certain circumstances in the twilight in cats, dogs, sheep, and in general in all animals having a tapetum. But whether or not a phosphoric light sometimes proceeds from the retina or choroid, has not as yet been fully ascertained. There are many examples of a luminousness in the dark having been observed in the human eye, yet it wants the tapetum. Probably, as Treviranus remarks, such cases may be of a pathological nature.—*Edinburgh New Philos. Journal.*

### PATHOLOGY.

*Bulimia.*—Ruysch gives an instance of bulimia, which was connected with a dilatation of the pylorus, in consequence of which the food slipped through the stomach into the intestines before there was time for digestion to take place; and it is recorded by Lieutaud, that, upon opening the body of a patient who had died of a disorder in which a voracious appetite was a leading symptom, he discovered a preternatural termination of the ductus choledochus in the stomach. In this case, the bile effused into the stomach seems to have kept up a constant irritation, by which the ingesta were expelled before digestion took place.—Wadd; *Quarterly Journal of Science.*

*Destruction of a Portion of the Spinal Cord.*—A man, whose case is related by Mr. Copeland, had paraplegia, dysuria, obstinacy of the bowels, and a feeling of tightness across his belly, as if a broad band had been tightly bound round it. His health had been declining for more than a year, and the commencement of his complaints was ascribed to having violently sprained his back in lifting a heavy weight. After being confined to bed with perfect paraplegia for three months, he died of gangrene of the nates.

On dissection, no disease could be discovered in the vertebræ; the spinal cord was entirely wanting for more than two inches. The membranes, which there formed an empty bag, were unusually vascular, and much thickened.

On the other hand, Ollivier found four inches of the cord entirely wanting in a child, aged eight years, who died of extreme marasmus, with caries of the vertebræ, but without loss either of sensibility or motion of the limbs.

Velpeau has described several cases in which, in connexion with caries of the vertebræ, the cord was completely destroyed for the space of several inches; the patient having died of gradual marasmus, without any appearance of paralysis.

In Magendie's Journal a case is described, in which the cord had become quite liquid, through two-thirds of the dorsal region and one third of the cervical. The arms were paralytic without loss of sensibility, but the legs were not affected

Ollivier has also observed in two cases a remarkable wasting or diminution of the size of the cord. The one was in an old man, without any particular symptoms; the other in an idiot, with permanent contraction and wasting of the limbs.—Abercrombie *on the Brain.*

*Inflammation of the Veins in general, and Inflammation of the Veins of the Uterus.* By Dr. Dance.—The common causes, such as punctures, laceration of the veins, &c. which produce inflammation of the veins generally, excite an inflammation which is confined to the part, and is simple in its nature: but, when the inflammation extends, attacks the internal coat of the vein, and pus is secreted, the disease assumes a dangerous character.

The general symptoms of inflammation of the veins, Dr. Dance divides into three orders: 1st. Those which are local and unattended by fever; 2d, the general phenomena of inflammation, according to its extent and intensity; 3d, the phenomena indicating the mixture of pus with the blood, and the development of various diseases in consequence.

Dr. Dance says, that the inflammation always extends in the same direction that the blood takes in the circulation, and assigns as the cause the transportation of pus. He considers the internal coat of the veins as a mucous membrane, and, when considerably inflamed, that pus is secreted. The pus thus secreted, says Dr. Dance, unites with the blood, is carried into the circulation, and is the cause of many remarkable diseases in distant parts; such as abscesses in the brain, liver, spleen, articulations, and, above all, the lungs; which show themselves in one or more of these parts after inflammation of the veins has existed, and pus has entered the circulation. He says, we cannot attribute the development of such diseases to any other cause than a mixture of pus with the blood, although its presence may not be capable of being demonstrated; and he thinks that this opinion offers the greatest probabilities, if we consider, 1st, the rapidity with which such diseases as mentioned above show themselves; 2d, the peculiar phenomena which belong to them; 3d, the appearance of the diseases immediately after inflammation of the veins; and lastly, the resemblance which the symptoms have to those of typhus, &c. Besides, says he, experiments have been made on living animals, in which the veins have been injected with putrid matter, and the symptoms that followed were analogous to those which present themselves in some cases of inflammation of the veins.

*Inflammation of the veins of the uterus* takes place only after delivery; it often extends to the substance of this organ: hence inflammation of the uterus; it may, however, be secondary to the inflammation of the uterus. Inflammation of the veins of the uterus is frequently confined to one side of the organ only: this probably depends on the attachment of the placenta to the uterus. It sometimes takes place in a manner which seems to be epidemic; an observation that singularly corresponds with that made by ancient authors relative to cases of severe fevers of recently delivered women; and Dr. Dance thinks these fevers were nothing more than inflammation of the veins of the uterus, complicated by purulent absorption.

Suppurations within, or external to, the joints, have been more commonly found after inflammation of the veins of the uterus, than after inflammation of the veins in general.

Dr. Dance says it would be desirable to know the characteristic or pathognomic symptoms of inflammation of the veins of the uterus, that it may be distinguished from inflammation of the uterus itself: but he does not know them.

To these propositions Dr. Dance adds some observations upon the treatment of inflammation of the veins generally, and gives the preference to the antiphlogistic plan in the first stages of the disease. He terminates by asking if it would not be rational, 1st, to employ compression above the point where the vein is inflamed, and before pus is secreted, to prevent the pus entering into the circulation? 2d. To wash the wound carefully when a vein is inflamed, to prevent the contact of pus with the internal membrane of the vein? 3dly. To inject emollient liquids into the cavity of the uterus after delivery? 4thly. In the third stage of the malady, when pus has mixed itself with the blood, and bleeding is no longer practicable, ought not antiseptics to be employed?*—*Nouvelle Bibliothèque Médicale.*

---

### PRACTICAL MEDICINE.

*Chronic Ptyalism.*—A case of this kind is related by SOUQUET, which was cured by the mastication of canella bark, and by swallowing the juice of it. For nine years and a half the patient had discharged from the mouth five pints of saliva daily. He had never had syphilis, nor had he taken mercurial medicines of any kind.

---

*Sulphur employed as a Preventive of Measles.*—In a family of four children, two took the flour of sulphur night and morning, and were entirely preserved from the contagious influence of the disease, although they continued to live in the same atmosphere, and were allowed to communicate freely with the other children who had the disease. Two of five adults, who lived in the same house, contracted measles: one had before had the disease. They had employed no precautionary means. In another family, one child had measles. Three other children were not separated from the patient: they took, night and morning, sulphur mixed in sugar, and escaped the disease. The dose of the sulphur should be from two to six or eight grains, according to the age.

In another case, an infant took the sulphur as soon as the disease had clearly manifested itself in his brother. In eight days, however, the measles appeared, but the malady ran so favorable a course that it was probable the preservative effects of the remedy had some influence. Four other children were treated in a similar manner: they were designedly exposed to the contagion, but entirely escaped.

It is not presumed that a confident opinion can be hazarded from so small a number of facts. They are sufficient, however, to engage the attention of the profession.—*Recueil de la Société Medicale de Tours.*

---

*Effects of Acupunctuation in a Case of Paralysis, which had existed for seven years.*—A young woman, twenty-two years of age, fell, about seven years ago, from a height of about four feet from the ground, on her back. After the accident she suffered great pain in the lumbar region, which continued. Three weeks after the fall she was attacked with peripneumonia, from which she soon recovered; but about the same time the lower right extremity became paralytic, in which she had great pain, and also continual pain in the loins. Baths and frictions were tried to restore the use of the limb, but without effect: its muscular power daily diminished, and she could not walk without the support of two sticks. In this state she continued for seven years after the accident, when she came into hospital to be cured of prurigo.

During her stay in the hospital, she witnessed the good effects of acupunctuation in several cases of paralysis, and therefore solicited M. TROUVÉ to try its effects in her case. He introduced four needles in the lumbar region, and

* The opinions of Mr. ARNOTT, as laid before the Medical and Chirurgical Society, in a very elaborate paper, are similar to those of Dr. DANCE; but the views of the former are much more extensively illustrated by cases.—EDITORS.

after the lapse of two hours withdrew them. The patient raised herself, and walked without support, which she had not been able to do for some years. The pain in the loins was greatly relieved, but the pain in the paralytic limb was more violent, and, as she expressed herself, "it seemed as if the disease had fixed itself entirely in the posterior part of the thigh." In the evening of the same day three more needles were introduced in the superior and back part of the thigh, in the tract of the sciatic nerve. They were withdrawn three hours after their introduction, and the pain of the thigh was completely removed. The pain in the leg, however, was more violent than before: two needles, therefore, were introduced into the calf of the leg. The pain immediately ceased, and the patient walked about without support. She still complained of pain in the foot: two needles were introduced, which removed it entirely, and the patient was considered as well.—*Archives Générales.*

---

*On the Employment of the Acetate of Ammonia in Uterine Diseases.* By M. Patin, D.M.P.—In the twelfth volume of the Archives Générales, page 651, M. Cloquet related a case of difficult menstruation, which was completely cured by the acetate of ammonia. This case attracted the notice of M. Patin, and led him to try this remedy in other cases of uterine disease. M. P. has related five interesting cases; and if experience should prove the truth of his observations in the employment of the acetate of ammonia in these diseases, the medical world will be much indebted to him. We shall give an outline of these cases, and shall state every thing that has direct relation to the subject.

Case I.—A married woman, aged thirty-four, whose health had been partly deranged in consequence of frequent returns of menorrhagia, and who had cancer of the neck of the uterus, as well as symptoms of phthisis, applied to M. Patin for relief of the dreadful sufferings which she experienced at the approach of the period of menstruation. The cancer of the uterus had existed two years before he saw her: lancinating pains, deep ulcerations, fetid discharge filled with flakes of matter and clots of black blood, and the neck of the uterus descending almost as low as the orifice of the vagina, were the symptoms that characterized this dreadful disease. All these symptoms increased considerably five or six days before the period of menstruation, which were relieved only by an abundant flow of the menses, which produced great exhaustion.

M. Patin administered forty drops of the acetate of ammonia, three days before the expected period of menstruation: it diminished the pains greatly. The menses appeared on the sixth day after the use of the medicine: they were abundant, but not so considerable in quantity as before.

Whenever the violent pains were felt, or when there was any appearance of hemorrhage, the patient took thirty or forty drops of this medicine, during the interval of menstruation, in a glass of water, which arrested the pains, and diminished the hemorrhage.

The next time menstruation took place, it was not attended with so much pain; the quantity of the discharge was not so great as before; and it continued about the same time as when in health. The neck of the uterus, by examination, was found to be less in size and much shorter; the ulcerations had a better appearance, and seemed disposed to heal; the discharge was not so fetid, and it did not contain so many flakes of matter. The patient could sit and walk without pain, which she had not been able to do for a very long time before.

M. Patin concludes this case by saying that he should not have been without hopes that a cure of the cancerous disease might have been effected, if the patient had not laboured under phthisis. She left Troyes as soon as she was able to travel, and he was therefore deprived of the opportunity of stating the result of her case.

Case II.—A girl, nineteen years of age, during four or five days which preceded menstruation, suffered pain and a sense of weight in the pelvis, pain of the head, nausea, frequent vomiting, restlessness, &c. These symptoms continued till the flow of the catamenia, which lasted about three days, and in quantity was rather small. The acetate of ammonia, given in the dose of thirty drops twice in the twenty-four days, the second day of the coming on of the above symptoms, freed the patient almost immediately of all her sufferings. The symptoms returned the third or fourth day, and another dose removed them entirely.

She has continued this treatment for three months, and menstruation is no longer painful to her; but the discharge, whilst she was taking the medicine, diminished in quantity, and continued for a day and a half only. It was therefore thought advisable to leave off the acetate of ammonia; which she did, and in a short time the discharge was as abundant as before, and continued to flow its usual length of time, without being attended by pain.

Case III.—A young woman, twenty-five years of age, who menstruated regularly, but suffered previous to the time and during the period of the discharge. Besides the local symptoms, she had a dry cough and great oppression at the chest. She took the acetate of ammonia as in the preceding case, and the result was the same: the prompt removal of the pains and a temporary diminution of the discharge.

Case IV.—A lady, aged thirty-two, of a nervous temperament, who menstruated at the age of twelve, and who had enjoyed good health, and whose periodical discharge had been very regular until domestic troubles had deranged her general health, consulted M. Patin respecting her irregularity of menstruation. She had borne three children in the space of four years; had suffered much from menorrhagia after marriage; and during the last confinement had an attack of peritonitis, which had nearly destroyed her. After recovering from these attacks, and enjoying twelve months' comparative good health, other domestic troubles arose, which had the effect of bringing on menstruation twice a month: the quantity of the discharge at each time was considerable, and it continued to flow for many days, leaving scarcely more than four or five days' interval between the termination of one period to the commencement of the other. This state was attended by a cough, oppression of the chest, loss of appetite, nausea, and loss of strength.

This was her state when she consulted M. Patin. He prescribed for her the acetate of ammonia in the dose of fifteen, increased to twenty-five drops morning and evening. The menses gradually diminished in quantity, and at the end of three months continued to flow for four days only. The other symptoms also disappeared, except the cough, which continued, but in a much less degree. Six months after this treatment menstruation took place regularly, and the general health was in a great degree restored.

Case V.—A woman, aged thirty-seven, of very irregular habits, menstruated at ten years of age, and married at twelve. Her catamenia had been regular, very abundant, and continued to flow for ten or twelve days. After marriage, leucorrhœa came on. She miscarried six successive times; yet notwithstanding so many causes of debility, she had remarkably good health until she was thirty-seven years of age.

In August and September, 1827, menorrhagia came on; a copious discharge of the menses continuing for seventeen days, attended with a dry cough and oppression at the chest. In October she did not menstruate; but, from November until the February following, she had almost constant discharges, which were sometimes considerable in quantity; and, during these discharges, a dry cough and oppression at the chest, loss of appetite, nausea, frequent vomitings, and intense heat of the genitals, which extended over the lower part of the abdomen, were the accompanying symptoms. In the month of February, the discharge did not make its appearance for twenty days. During March and April, it was so great as to wet through eight or ten cloths doubled in the course of the day.

In May 1828, when she consulted M. Patin for the first time, her state was most deplorable: she was extremely thin, her countenance of a yellowish hue, her eyes dull, her skin hot and dry, her pulse frequent and small, a dry and troublesome cough, oppression at the chest; a considerable discharge of sanguineous fluid from the uterus, with clots of blood; a burning heat in the pelvis, great pain in the right lumbar region, particularly during the emission of urine; loss of appetite, obstinate costiveness, pain in the region of the stomach, nausea and frequent vomitings of mucous matter; great thirst; lower part of the abdomen tense and painful. The neck of the uterus cold, lax, much enlarged, and very sensible to the slightest touch, the least pressure occasioning a discharge of blood from it.

A blister to the arm, astringent lotions to the lower part of the abdomen, and other means, were used for five days, without effect. By taking forty drops of the acetate of ammonia three times a day, keeping in the horizontal position, and remaining perfectly quiet, the chief symptoms were removed in four days, a slight discharge from the uterus only remaining: for this she was directed to use an injection of port wine and infusion of roses, which arrested the discharge in a short time.

It is remarkable that, before she took the acetate of ammonia, whilst she was suffering in the manner above described, her violent desire for coition was not in the least abated; but the acetate of ammonia seemed to have the effect of counteracting this morbid passion, and, as she expressed herself, of "freezing and depriving her of a pleasure which was more dear to her than life."

She had recovered her usual strength, and was almost perfectly restored to health, when, on the 1st and 2d of June, she imprudently walked a great distance, which brought on menorrhagia again, and all the symptoms above described. She used the astringent lotion for three days, without consulting any medical person, but it only increased the symptoms. On the 4th of June she again consulted M. Patin, who prescribed sixty, and afterwards seventy, drops of the acetate of ammonia four times a day. The effect of the remedy was instantaneous, as in the evening of the same day she was very much better, and at the end of forty-eight hours had no other symptom than a slight sanguineous discharge, which was soon arrested by the vinous injection. From the 6th of June to the 6th of July, the patient has had none of the above symptoms, and seems to be perfectly recovered.

M. Patin concludes by saying that these cases, and others, have proved to him that the acetate of ammonia is applicable,

1st. In cases of painful menstruation; although with some reserve, as it diminishes the quantity of the discharge.

2d. In cases of menorrhagia, in which he has obtained the most decided benefit from the use of it.

3d. In cases of cancer of the uterus, in which it acts at least as a powerful palliative.

4th. In cases of nymphomania; though he acknowledges that he has but one single fact (as given in Case V.) to support this conclusion.

M. Patin considers the acetate of ammonia to have a special action on the uterus, and this action to be decidedly sedative; therefore, says he, reasoning by analogy, it may be of use in cases of threatened abortion, in inflammations of the uterus and ovaries, &c. The largest dose of this medicine that M. Patin has given in uterine diseases has been seventy drops: this dose, he says, produces stupor and a species of intoxication, which lasts some minutes; though in other diseases he has given as much as five ounces in the space of twenty-four hours.

---

### SURGERY.

*Aneurism of the Carotid Artery cured by* VALSALVA's *Plan.*—The aneurismal swelling was seated on the left side of the neck, and extended from the thyroid gland to the clavicle. It was larger than a hen's egg, and pulsated very strongly. The integuments covering it were of a natural colour. It was entirely cured by a long continuance of the strictest regimen, consisting of weak broths, bread, vegetables, acid drinks, bodily tranquillity, repeated bleeding, the exhibition of digitalis and of laurel-water. Ice was also frequently applied to the tumor. Compression could not be borne. The patient occasionally suffered from attacks of difficult deglutition.

At the time this case was recorded, four years had elapsed, and he remained perfectly well.—*Hufeland's Journal.*

---

*Ligature of the external Iliac Artery.*—M. RICHERAND lately presented to the Royal Academy of Medicine a patient, upon whom he had performed the operation for aneurism. No untoward symptom followed. On the day after the ligature was applied, no numbness was felt in the limb, and the man was anxious to rise from his bed. The ligature came away on the twenty-fifth day after the operation, and on the fortieth the wound was completely cicatrized. M. R. conceives that the favorable progress of the case depended on the particular manner in which the operation was performed. The peculiarity, upon which much stress appears to be laid, was that, after having detached the peritoneum, especial care was taken not to separate the vessel to a greater extent than was absolutely necessary for the application of the ligature. This precaution is doubtless of much consequence, but we apprehend that English surgeons never neglect to adopt it in all cases of aneurism.

---

*Fungus Hæmatodes cured by Alum and the Red Oxyd of Mercury.*—Madame Brick, twenty-six years of age, had suffered in infancy from severe scrofulous affections. She had had ulcers in the neck and other parts of the body. Her health had latterly been good. In 1820 she consulted Dr. SCHUTTE respecting a small tumor on her left cheek, immediately above the ear. It was about the size of a nut, and resembled an encysted tumor. Although it was moveable, it appeared to adhere partially to the skin. Seveial small dilated vessels were observed on the skin which covered it. On the summit of the tumor were several warty excrescences. The tumor had been caused by a blow which had been inflicted some weeks before, and had not been interfered with from a hope that it would spontaneously disperse.

Dr. S. did not again see the patient until January 1823. The tumor was now the size of the fist. It was still moveable at the base, but the skin which covered it was of a bluish tint. The vessels which ramified over it were not increased in size or number. The tumor was elastic, and not hard. It appeared as if it contained pus.

In April, the patient observing a point in which a fluctuation was perceptible, opened it with a pin, for the purpose of discharging the supposed matter. Violent hemorrhage succeeded, which could only be arrested by the application of a very tight bandage. Two days after, a still more formidable bleeding took place, which threatened to destroy her. She lost more than two pints of blood; and the small opening which had been made had enlarged to the size of a sixpence. The immediate danger was obviated by the application of lint, rosin, gum arabic, and a bandage. A strengthening regimen soon restored her health.

The dressings were removed five days afterwards. The wound had still enlarged. Hemorrhage again took place, and was arrested by the same means.

In three days the dressings were renewed. The wound was now two inches and a half in diameter. The hemorrhage was more violent than ever. Calcined alum was now applied, very finely powdered.

Upon the next examination, two days afterwards, there was a very slight discharge of blood, which appeared to flow from many small openings over the whole surface of the wound. The interior of the wound resembled a fine moistened sponge. It was of a bluish white colour, in some parts rather red.

No alteration took place for three days. A sixth part of the red oxyd of mercury was now added to the alum. The wound was dressed every day, and each time the quantity of the oxyd of mercury was increased.

Under this treatment, the morbid substance gradually diminished. When the upper half of the fungus had disappeared, a compact substance was seen at the bottom of the wound, which resembled, both in colour and consistence, the medullary substance of the brain. A small quantity of blood was still discharged from the substance, but in a much less quantity than from the fungoid substance. This encephaloid matter was also removed by equal parts of alum and red precipitate, and the cure was completed in April 1824.

Quinine and other bitters were given internally throughout the treatment of the case.—*Graefe's Journal.*

---

*Remedy for Sore Nipples.*—We have frequently found the following simple remedy very efficacious: it is to be applied after suckling.

℞. Pulv. Acaciæ ℥ss.; Aluminis gr. v. M. diligentissime ut fiat pulvis, cujus inspergatur pauxillum super mamillas pro re natâ.

We do not remember to whom we are indebted for this prescription.

*Gleet cured by an Injection of Sea Water.*—An obstinate case of gleet is recorded in the eleventh volume of the Edinburgh Medical Journal, by Mr. Fletcher, which was cured by an injection of sea water. The patient had laboured under the complaint for two years. It was removed in ten days.

---

### MIDWIFERY.

*A Case of Extra-uterine Abdominal Pregnancy.* By Dr. Mitivié.—This is a very interesting and curious case, not only because it presents a considerable degree of ossification, of development, and conformation of the cranium of a fœtus, but because it existed in the cavity of the peritoneum of a woman seventy-seven years of age; to whom it appears not to have occasioned the least inconvenience.

A woman, who had had several children, died, aged seventy-seven, in l'Hospice de la Salpétrière. The post-mortem examination presented an irregular body in the cavity of the abdomen, floating, and attached only by cellular tissue to the mesentery and a portion of the small intestines. The peritoneum, uterus, and its appendages, and in short all the viscera, appeared to be healthy.

The tumor was easily removed from the abdomen, and was found to be a fœtal skeleton enveloped by a thin membrane, which was nearly diaphanous. The length of the tumor was two inches; it was divided into two unequal portions by a kind of neck; the largest portion contained the cranium, and the smallest portion the trunk, of the fœtus. On examining the skeleton, the cranium was found to be ossified, and not very ill formed; its size was, in the anterior posterior diameter, one inch eight lines and a half; transversely, one inch four lines; vertically, one inch. All the different bones of the cranium could be easily distinguished: the fontanels had disappeared; all the sutures were united. The orbits were formed, and the superciliary ridges perfect; but the base of the os occipitale was not perfectly ossified, and there were no ossa maxillaria. The cranium was united to the trunk by fibro-cartilaginous bands, and probably by articulating surfaces, which could not be ascertained, as it was desirable to preserve the little skeleton as entire as possible.

The trunk was bent a little forward, and surrounded by a sort of layer of cellular tissue: it presented the rudiments of the vertebral column, sternum, and ribs. There were no lower extremities, but on the sides of the thorax there were fragments of the bones of the arms.

On dividing the skeleton perpendicularly through the median line, the cranium appeared well formed: its walls were about half a line in thickness; they were lined by the dura mater. A yellow gelatinous fluid, without any distinct organization, but surrounded by a thin membrane, filled its cavity.

The cervical vertebræ were made up of several pieces, which being irregularly disposed, each vertebra could not be easily distinguished. The dorsal vertebræ consisted of osseous rings, and were more easily traced. The lumbar vertebræ consisted also of rings of osseous matter, and were easily distinguished; as was also the sacrum.

The thorax and abdomen appeared to form but one cavity. The thorax was empty superiorly, where the pleura was distinctly seen upon the ribs; inferiorly, it contained a mass of grayish yellow matter, resembling fat. This mass presented various folds, and was without doubt the remains of the viscera. In the centre of this mass there was a kind of brownish kernel, extending the whole length of the vertebral column, provided with a small cavity. This was probably the heart and the aorta: but it was difficult to ascertain this point.

Blood-vessels were seen about the head and thorax of the fœtus, and without doubt these vessels were furnished through the means of the cellular membrane which connected the tumor to the mesentery and small intestines; although, on close examination, the cellular membrane itself presented no vessels.—*Archives Générales.*

### MISCELLANEOUS.

*Communication of Disease by Leeches.*—It is important to bear in mind that leeches, which had been applied first to a syphilitic patient, and afterwards to an infant, communicated the disease to the latter.—*Westphaelischer Anzeiger.*

*Preservation of Leeches.*—A new vessel of deal, large enough to contain sufficient water for five hundred leeches, is to be furnished with a stopcock, to draw off the water. It is to be half filled with the mud from the lake or pond whence the leeches have been taken, and two or three roots of the Florence iris (Calamus Aromaticus) are to be set in the mud. The leeches like this plant. The usual precautions as to temperature, frequent change of water, &c. are to be taken. The water is to be changed slowly, and the fresh water added by means of a funnel descending to the bottom of the vessel. This method has been found preferable to all others tried at the hospital of Bamberg.—*Bull. Univ.*

*Account of an Idiot of an Herbivorous Habit.* By Dr. FRANCOIS.—A girl, named Roger, twenty years old, is a perfect idiot. Her physical development was tardy, although at present she is very strong: she was three years old before she could walk. She has never spoken; her wants and her desires are expressed by cries which much resemble grunting; she is not deaf, obeys when she is ordered, and appears to be of a peaceable disposition. When she is contradicted, she expresses her anger by scratching the root of her nose. When seated or lying down, her head and hands are in constant motion, and apparently without any design. She tears every thing that falls into her hands. She is of the middle size, and thick set; her skin is white, eyes blue; her forehead prominent and protruding; her mouth wide, and her lips very thick; her countenance is healthy, but her face is without expression. Her step is uncertain, and resembles that of a person scarcely awake.

This unfortunate girl moves about voluntarily on her hands and knees; and in this position searches about, smells at every thing, and puts what she finds into her mouth. She seems to prefer finding her food, rather than that it should be given to her. She satisfies the wants of nature wherever she may chance to be, without shame or scruple. The food she prefers is trefoil, clover, and groundsel; next to these things, raw meat and the entrails of animals. She dislikes every thing that is cooked, and eats bread only for want of something better. She tears up grass, which she makes into a kind of bundle, and places it between the molares on one side of the mouth; then, without using the other teeth, she grinds it by moving her jaws horizontally. She likes wine very much, but does not drink it in the common way; being accustomed, without doubt, to quench her thirst in rivulets, she therefore laps and sucks up all liquids. She cannot distinguish the sexes. Abandoned by her parents, she has imbibed the practices and allurements of the animals with which she has lived. Her father declares that she knows her way sufficiently to return home even at half a league distance. She was three years old when she first showed a taste for raw meat. The entrails of a rabbit having been thrown into a courtyard, the girl seized them, and disputed the possession of them with a dog. Passing almost all her time in the fields among beasts, example and hunger have taught her to feed upon grass.

This girl is to be placed in the Salpêtrière.—*Journal Générale.*

---

*Innocuous Nature of Putrid Exhalations.*—A committee has been engaged in France in examining the circumstances relative to the knacker's operations. His business consists in killing old worn-out horses, and turning every part of their body to account. The most singular results which the committee have obtained relate to the innocuous nature of the exhalations arising from the putrefying matter. Every body examined agreed that they were offensive and disgusting, but none that they were unwholesome: on the contrary, they appeared to conduce to health. All the men, women, and children concerned in the works of this kind had unvarying health, and were remarkably well in appearance and strong in body. The workmen commonly attained an old age, and were generally free from the usual infirmities which accompany it. Sixty, seventy, and even eighty, were common ages. Persons who live close to the places, or go there daily, share these advantages with the workmen. During the time that an epidemic fever was in full force at two neighbouring places, not one of the workmen in the establishment at Mountfaucon was affected by it. It did not appear that it was only the men who were habituated to the works that were thus favored; for when, from press of business, new workmen were taken on, they did not suffer in health from the exhalations.

In confirmation of the above observations, similar cases are quoted. Above two hundred exhumations are made yearly at Paris, about three or four months after death: not a single case of injury to the workmen has been known.

M. Labarraque has observed that the catgut makers, who live in a continually putrid atmosphere, arising from macerating intestines, enjoy remarkable health.

Similar circumstances were remarked at the exhumations of the Cimetière des Innocens.

Whatever disease the horse may have died of, or been killed for, the workmen have no fear, adopt no precautions, and run no risk. Sometimes, when strangers are present, they pretend to be careful; but, in private, really laugh at such notions. They handle diseased as well as healthy parts, always with impunity. They frequently cut themselves, but the wounds heal with the greatest facility; and their best remedy is to put a slice of the flesh about the wound.

On making inquiry of those to whom the horse skins were sent, and who, besides having to handle them when very putrescent, were more exposed to effects from diseases in the skin, they learnt that these men also, from experience, had no fear, and never suffered injury. Horse-skins never occasioned injury to those who worked them; but in this they differed from the skins of oxen, cows, and especially sheep, which sometimes did occasion injury though not so often as is usually supposed.—*Recueil Industriel.*

### INTELLIGENCE.

